# Interpretable gradient boosting machine model for predicting in-hospital mortality in sepsis-induced myocardial injury: a multicenter development, validation, and web-based clinical implementation

**DOI:** 10.3389/fcvm.2026.1737106

**Published:** 2026-07-01

**Authors:** Lina Chen, Qianru Yuan, Yitong Ma

**Affiliations:** Cardiac Center, The First Affiliated Hospital of Xinjiang Medical University, Urumqi, Xinjiang, China

**Keywords:** gradient boosting machine, in-hospital mortality prediction, machine learning, multicenter validation, sepsis-induced myocardial injury

## Abstract

**Background:**

Sepsis-induced myocardial injury (SIMI) is a life-threatening complication of sepsis, associated with high in-hospital mortality. Current risk prediction models for SIMI lack interpretability and multi-center validation, necessitating advanced analytical approaches to improve risk stratification and clinical decision-making.

**Method:**

In this study, LASSO regression for feature selection, eight machine learning algorithms were evaluated, including Gradient Boosting Machine (GBM), XGBoost, and Logistic Regression. Model performance was assessed via AUC, sensitivity, specificity, and calibration curves. SHapley Additive exPlanations (SHAP) were used to interpret feature contributions, and recursive feature elimination optimized the model for clinical usability.

**Result:**

The GBM model demonstrated optimal performance with an internal validation AUC of 0.751 (95% CI: 0.614–0.867), outperforming other algorithms (e.g., SVM: 0.733, LR: 0.730). External validation in eICU and the Chinese cohort yielded AUCs of 0.924 and 0.703, respectively, confirming generalizability. Key predictors identified by SHAP included APS III Score, Hypertension, Albumin, Diabetes, SOFA Score, ALT, RBC, and Lactate. A simplified model with five variables (age, SOFA score, APS III score, albumin, ALT) achieved an AUC of 0.789 and was deployed on a user-friendly platform (https://anyuanning.shinyapps.io/SIMI/), enabling real-time risk assessment for clinicians.

**Conclusion:**

This multicenter study developed an interpretable predictive model to predict in-hospital mortality in patients with sepsis-induced myocardial injury (SIMI) and deployed it on a user-friendly platform, potentially improving patient outcomes through early risk assessment.

## Background

Sepsis, a life-threatening organ dysfunction caused by a dysregulated host response to infection, is characterized by high mortality. Approximately 18 million severe sepsis cases occur globally each year, with mortality rates ranging from 30% to 70%. As a critical complication of sepsis, sepsis-induced myocardial injury (SIMI) affects up to 50% of septic patients and is strongly associated with poor prognosis (1, 2). A Study has shown that septic patients with cardiac dysfunction exhibit a mortality rate as high as 70%, significantly higher than the 20% mortality rate observed in those without cardiac dysfunction (3).

SIMI is primarily characterized by impaired cardiac contractility, elevated cardiac biomarkers (such as cardiac troponin T, cTnT), and echocardiographic abnormalities. Its pathological process is often exacerbated by systemic inflammation, oxidative stress, and mitochondrial dysfunction. Research indicates that the in-hospital mortality rate of SIMI patients reaches 35%, while the 1-year mortality rate soars to 51% (4). Despite notable advancements in sepsis management, early risk stratification for in-hospital mortality in SIMI patients remains challenging due to the heterogeneity of clinical presentations and the lack of reliable, condition-specific predictive tools.

Current risk stratification for SIMI relies predominantly on traditional scoring systems, such as the Sequential Organ Failure Assessment (SOFA) and Acute Physiology Score III (APS III) (5). These systems evaluate disease severity by quantifying multi-organ dysfunction and physiological derangements, providing valuable prognostic insights but demonstrating limited predictive efficacy for in-hospital mortality in SIMI populations. A core limitation lies in their inability to capture dynamic interactions between clinical variables or model the nonlinear relationships inherent in complex critical care data. Additionally, although traditional biomarkers like cTnT are sensitive for myocardial injury, their ubiquitous elevation in sepsis reduces their discriminative value in SIMI cohorts, underscoring the need for multidimensional risk assessment frameworks. Most existing clinical tools, based on conventional statistical methods (e.g., logistic regression), struggle to model complex nonlinear associations and lack validation using multi-center datasets. Furthermore, the “black-box” nature of many models limits their practical application in clinical decision-making, as their underlying mechanisms remain opaque to clinicians (6).

In summary, this multicenter study aims to address critical unmet needs by developing an interpretable ML model for predicting in-hospital mortality in SIMI patients. By deploying a user-friendly online platform for real-time risk assessment, this study bridges the translational gap between predictive analytics and clinical decision-making, offering a scalable tool to mitigate the substantial burden of SIMI-related mortality.

## Method

### Data sources

This study derived data from two public databases and one dataset from the First Affiliated Hospital of Xinjiang Medical University. The former includes the Medical Information Mart for Intensive Care-IV (MIMIC-IV) and the eICU Collaborative Research Database (eICU). MIMIC-IV represents an extensive publicly available database, which encompasses the clinical details of adult patients who are 18 years old or above and were admitted to the intensive care unit (ICU) of a premier tertiary hospital in the United States during the period from 2008 to 2019 (7). eICU database comprises 200,859 inpatient data originating from ICUs and step-down units within 208 hospitals scattered across the United States (8). We have successfully completed the Collaborative Institutional Training Initiative (CITI) exam (Record ID: 66254112) and obtained the permission to access this database. The Beth Israel Deaconess Medical Center and the Institutional Review Board of the Massachusetts Institute of Technology have approved the use of these data. Moreover, the two databases, MIMIC-IV and eICU, did not require ethical review. The dataset from the First Affiliated Hospital of Xinjiang Medical University has obtained approval from the Ethics Committee (Approval Number: K202501-02). As this is a retrospective study, we retained patient personal information and obtained a waiver of informed consent. All procedures in this study were conducted in accordance with the Declaration of Helsinki and its subsequent amendments.

### Study population

**Figure 1:**
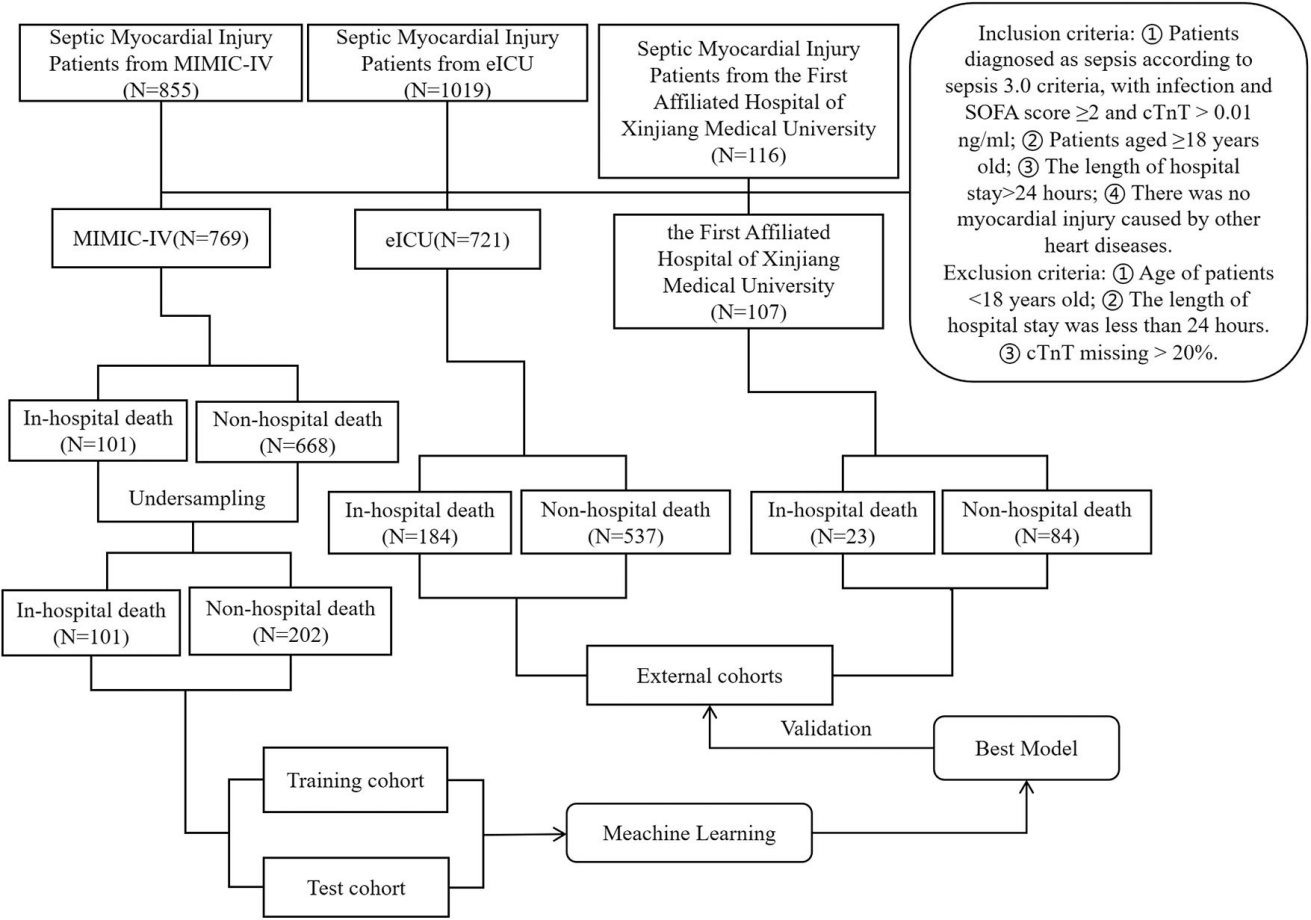
Flowchart for extracting patient screening information from databases.

This study included 769 patients diagnosed with septic myocardial injury in the MIMIC-IV database from 2008 to 2019, 721 patients diagnosed with septic myocardial injury in the eICU-CRD database from 2014 to 2015, as well as 107 patients diagnosed with septic myocardial injury from the First Affiliated Hospital of Xinjiang Medical University. The specific process is shown in Figure 1.

Inclusion criteria: ① Patients meeting the Sepsis-3.0 criteria for sepsis (infection plus SOFA score ≥2) and with cTnT > 0.01 ng/mL (above the upper normal limit) conform to the diagnostic criteria in the Emergency Experts Consensus on Adult Sepsis-Related Myocardial Injury and/or Cardiac Dysfunction. ② Patients aged ≥18 years old; ③ The length of hospital stay > 24 h; ④ There was no myocardial injury caused by other heart diseases. Exclusion criteria: ① Age of patients <18 years old; ② The length of hospital stay was less than 24 h. ③ cTnT missing > 20%.

To exclude myocardial injury attributable to causes other than sepsis, we relied on the database's existing diagnosis of SIMI, which inherently required the absence of acute coronary syndrome or other primary cardiac disorders. Additionally, all included patients underwent echocardiography, and those with significant structural heart disease that could independently explain myocardial injury (e.g., severe valvular disease, cardiomyopathy) were excluded. No additional exclusion based on specific ICD-10 codes, electrocardiographic thresholds, or hemodynamic cutoffs was performed, because these data were already incorporated into the SIMI diagnosis.

### Variable selection

This study used Structured Query Language (SQL) and Navicat Premium software to extract data from the MIMIC-IV database and the eICU database.

Demographic characteristics included age, gender, Weight. Comorbidities included Cardiovascular diseases (CAD), Heart failure, Atrial fibrillation, Hypertension, Diabetes, Chronic Kidney Disease (CKD), Chronic pulmonary, Liver disease. Treatment measures included Steroid, Vasopressor, Ventilation, and Renal replacement therapy (RRT). Physiological status includes Systemic Inflammatory Response Syndrome Score (SIRS Score), SOFA Score, APS III, Simplified Acute Physiology Score II (SAPS II Score), Heart Rate (HR), and Temperature. Laboratory indicators included PaCO2, PaO2, cTnT, Lactate, White Blood Cell Count (WBC), Neutrophils, Lymphocytes, Hematocrit, Red Blood Cell Count (RBC), Platelet Count (PLT), Albumin,Serum Creatinine (Scr), Bilirubin, BUN, Glucose, Alanine Aminotransferase (ALT), Aspartate Aminotransferase (AST), Prothrombin Time (PT), Partial Thromboplastin Time (PTT), International Normalized Ratio (INR).

Outcome variables: The primary outcome was all-cause mortality. The secondary outcomes included in-hospital mortality (the situation of patients dying during hospitalization) and ICU mortality (the situation of patients dying during ICU stay).

### Data preprocessing and feature selection

All analyses were performed using Python 3.8 and R 4.0.3. For variables with a missing rate less than 20%, imputation was performed using the “Mice” package, while variables with a missing rate greater than 20% were removed, see Supplementary Figure S1 for details. Continuous variables were presented as medians with interquartile ranges (IQR), and categorical variables were expressed as frequencies and percentages. The Mann–Whitney *U*-test was used to compare continuous variables, while the chi-square test was employed for categorical variables. Statistical significance was set at a *P*-value < 0.05. Feature selection was performed using Least Absolute Shrinkage and Selection Operator (LASSO) regression to identify the most relevant predictors of in-hospital mortality. All continuous variables were standardized to have a mean of 0 and a standard deviation of 1 before performing LASSO regression. The optimal lambda value was determined through 10-fold cross-validation, resulting in the selection of 18 key variables, including age, gender, heart failure, hypertension, diabetes, chronic kidney disease (CKD), liver disease, vasopressor use, SIRS score, SOFA score, APS III score, cardiac troponin T (cTnt), lactate, red blood cell count (RBC), albumin, alanine aminotransferase (ALT), and partial thromboplastin time (PTT).

### Model development and validation

Eight machine learning algorithms were employed to construct predictive models: Logistic Regression (LR), Support Vector Machine (SVM), Naive Bayes (NB), Gradient Boosting Machine (GBM), AdaBoost, XGBoost, Multilayer Perceptron (MLP), and Random Forest (RF). The models were trained on 80% of the MIMIC-IV dataset, with the remaining 20% used for internal validation. Model performance was evaluated using the area under the receiver operating characteristic curve (AUC), sensitivity, specificity, F1 score, accuracy, and recall. External validation was conducted using the EICU dataset to assess the generalizability of the models.

### Hyperparameter tuning strategy

To ensure fair comparison and optimal performance, hyperparameter tuning was performed for all eight machine learning algorithms using 10-fold cross-validation on the training set. Grid search was employed to explore the hyperparameter space for each model, with the area under the AUC as the optimization metric. The hyperparameter grids were defined based on recommendations from the literature and preliminary experiments. For tree-based ensemble methods (GBM, XGBoost, Random Forest, AdaBoost), key parameters such as number of estimators, maximum depth, learning rate, and minimum samples split were tuned. For SVM, kernel type, regularization parameter C, and gamma were optimized. For logistic regression, regularization strength and penalty type were explored. For MLP, hidden layer sizes, activation function, and learning rate were tuned. For Naive Bayes, default parameters were used as it has limited tunable parameters. The final selected hyperparameters for each model are provided in Supplementary Table S1.

### Model interpretability

To enhance the interpretability of the models, the SHapley Additive exPlanations (SHAP) method was utilized. SHAP values were calculated to quantify the contribution of each feature to the model's predictions. A swarm plot was generated to visualize the impact of individual features on the model's output, and a force plot was used to illustrate the prediction process for specific patient cases. This approach allowed clinicians to understand the key factors driving the model's predictions and facilitated the identification of high-risk patients. Finally, we employed feature recursive elimination (RFE) for further variable filtering, aiming to develop a simplified model. RFEwas performed with GBM as the base estimator, using AUC as the core index, iteratively removing the lowest-contribution features, and terminating when the variable model achieved optimal comprehensive performance with clinical usability.

## Result

### Baseline characteristics

A total of 769 patients from the MIMIC-IV database were included in this study, among which 101 patients (13.13%) died in the hospital. In addition, according to the inclusion criteria, 721 patients were included from the EICU database, among which 184 patients (25.52%) died in the hospital, and 107 patients were included from the First Affiliated Hospital of Xinjiang Medical University, among which 23 patients (21.50%) died in the hospital. Supplementary Tables S2, S3 show the baseline characteristics of the patients in the MIMIC-IV and EICU databases, as well as the baseline characteristics of the patients in the MIMIC-IV and the database of the First Affiliated Hospital of Xinjiang Medical University. The results showed that there were significant differences in body weight, comorbidities, treatments, scores, vital signs, laboratory indices, and in-hospital mortality rates between the MIMIC-IV database and both the EICU database and the database of the First Affiliated Hospital of Xinjiang Medical University.

Table 1 presents the baseline information of all patients included from the MIMIC-IV database. Notably, patients who experienced in-hospital mortality had fewer comorbidities such as CAD, heart failure, hypertension, diabetes, CKD, and chronic pulmonary disease, and were less likely to receive vasopressor treatment but more likely to receive ventilation therapy. These patients also had slightly lower admission temperatures, lymphocyte counts, and albumin levels. Compared to patients who did not experience in-hospital mortality, these patients had higher SIRS scores, SOFA scores, APS III scores, and SAPS II scores, as well as elevated heart rates, lactate levels, bilirubin, and AST levels. There were no significant differences between the two groups in terms of weight, PaCO2, PaO2, cTnt, WBC, neutrophils, hematocrit, RBC, PLT, Scr, BUN, glucose, ALT, PT, PTT, INR, gender, atrial fibrillation, liver disease, steroid use, or RRT. All predictor variables, including laboratory indices and clinical scores (SOFA, APS III), were collected as the first baseline measurements within 24 h of ICU admission; SOFA and APS III scores were calculated based on these admission baseline data.

**Table 1:** Comparison of baseline characteristics between non-hospital death group and in-hospital death group.

Variables	Total (*n* = 769)	Non-hospital death (*n* = 668)	In-hospital death (*n* = 101)	*P*
Age	72.00 (61.00, 81.00)	71.00 (61.00, 81.00)	75.00 (65.00, 82.00)	0.023
Gender				0.121
Male	443 (57.61)	392 (58.68)	51 (50.50)	
Female	326 (42.39)	276 (41.32)	50 (49.50)	
Weight	77.00 (65.00, 90.50)	77.50 (65.70, 91.00)	72.00 (62.50, 86.70)	0.109
CAD				**0**.**002**
No	356 (46.29)	295 (44.16)	61 (60.40)	
Yes	413 (53.71)	373 (55.84)	40 (39.60)	
Heart failure				**<**.**001**
No	308 (40.05)	249 (37.28)	59 (58.42)	
Yes	461 (59.95)	419 (62.72)	42 (41.58)	
Atrial fibrillation				0.102
No	391 (50.85)	332 (49.70)	59 (58.42)	
Yes	378 (49.15)	336 (50.30)	42 (41.58)	
Hypertension				**<**.**001**
No	140 (18.21)	101 (15.12)	39 (38.61)	
Yes	629 (81.79)	567 (84.88)	62 (61.39)	
Diabetes				**0**.**003**
No	426 (55.40)	356 (53.29)	70 (69.31)	
Yes	343 (44.60)	312 (46.71)	31 (30.69)	
CKD				**<**.**001**
No	380 (49.41)	314 (47.01)	66 (65.35)	
Yes	389 (50.59)	354 (52.99)	35 (34.65)	
Chronic pulmonary				**0**.**024**
No	470 (61.12)	398 (59.58)	72 (71.29)	
Yes	299 (38.88)	270 (40.42)	29 (28.71)	
Liver disease				0.688
No	605 (78.67)	524 (78.44)	81 (80.20)	
Yes	164 (21.33)	144 (21.56)	20 (19.80)	
Steroid				0.063
No	383 (49.80)	324 (48.50)	59 (58.42)	
Yes	386 (50.20)	344 (51.50)	42 (41.58)	
Vasopressor				**<**.**001**
No	507 (65.93)	466 (69.76)	41 (40.59)	
Yes	262 (34.07)	202 (30.24)	60 (59.41)	
Ventilation				**<**.**001**
No	383 (49.80)	354 (52.99)	29 (28.71)	
Yes	386 (50.20)	314 (47.01)	72 (71.29)	
RRT				0.171
No	672 (87.39)	588 (88.02)	84 (83.17)	
Yes	97 (12.61)	80 (11.98)	17 (16.83)	
SIRS Score				**<**.**001**
0	9 (1.17)	9 (1.35)	0 (0.00)	
1	70 (9.10)	69 (10.33)	1 (0.99)	
2	165 (21.46)	151 (22.60)	14 (13.86)	
3	309 (40.18)	263 (39.37)	46 (45.54)	
4	216 (28.09)	176 (26.35)	40 (39.60)	
SOFA Score	4.00 (2.00, 6.00)	4.00 (2.00, 6.00)	6.00 (4.00, 9.00)	**<**.**001**
APS III Score	50.00 (40.00, 64.00)	47.00 (38.00, 60.00)	70.00 (55.00, 89.00)	**<**.**001**
SAPS II Score	39.00 (31.00, 49.00)	38.00 (30.75, 46.00)	50.00 (39.00, 63.00)	**<**.**001**
HR	87.00 (75.00, 100.00)	87.00 (75.00, 100.00)	89.00 (78.00, 109.00)	**0**.**040**
Temperature	36.60 (36.00, 37.10)	36.60 (36.10, 37.10)	36.30 (35.60, 37.00)	**0**.**025**
PaCO2	41.00 (36.00, 47.00)	41.50 (36.00, 48.00)	40.00 (35.00, 46.00)	0.242
PaO2	111.00 (70.00, 229.00)	110.50 (70.00, 225.25)	116.00 (74.00, 264.00)	0.266
Ctnt	0.14 (0.05, 0.49)	0.14 (0.05, 0.44)	0.15 (0.05, 1.01)	0.362
Lactate	1.60 (1.20, 2.40)	1.60 (1.10, 2.30)	2.10 (1.40, 3.40)	**<**.**001**
WBC	8.80 (6.50, 12.40)	8.80 (6.50, 12.20)	8.80 (6.40, 13.70)	0.483
Neutrophils	77.20 (65.60, 85.90)	76.65 (65.70, 85.20)	80.80 (64.00, 88.30)	0.291
Lymphocytes	14.20 (8.00, 23.90)	15.00 (8.80, 23.92)	10.30 (6.30, 21.30)	**0**.**004**
Hematocrit	12.00 (10.60, 13.60)	12.10 (10.60, 13.60)	11.70 (10.50, 13.60)	0.485
RBC	14.50 (13.70, 15.90)	14.40 (13.60, 15.80)	14.60 (13.80, 16.00)	0.153
PLT	234.00 (173.00, 305.00)	234.00 (173.75, 304.00)	232.00 (168.00, 312.00)	0.865
Albumin	3.60 (3.00, 4.00)	3.60 (3.10, 4.00)	3.10 (2.70, 3.80)	**<**.**001**
Scr	1.20 (0.90, 1.80)	1.20 (0.90, 1.80)	1.40 (1.00, 2.00)	0.097
Bilirubin	0.50 (0.30, 0.80)	0.50 (0.30, 0.80)	0.60 (0.40, 0.90)	**0**.**008**
BUN	26.00 (17.00, 39.00)	25.00 (17.00, 38.00)	30.00 (18.00, 43.00)	0.056
Glucose	6.83 (5.49, 9.60)	6.77 (5.49, 9.49)	7.44 (5.55, 9.82)	0.217
ALT	86.00 (66.00, 120.00)	86.00 (66.00, 120.00)	85.00 (64.00, 117.00)	0.722
AST	27.00 (20.00, 46.00)	27.00 (20.00, 45.00)	34.00 (22.00, 58.00)	**0**.**019**
PT	13.90 (12.90, 16.00)	13.80 (12.80, 15.60)	14.40 (13.10, 18.00)	0.064
PTT	29.40 (25.90, 34.70)	29.20 (25.80, 34.12)	30.40 (26.70, 37.20)	0.051
INR	2.00 (1.80, 2.30)	2.00 (1.80, 2.30)	2.10 (1.80, 2.30)	0.877

Bold values denote variables with statistically significant intergroup differences (*P* < 0.05). Continuous variables are presented as median (interquartile range); categorical variables as *n* (%).

### Feature selection

**Figure 2:**
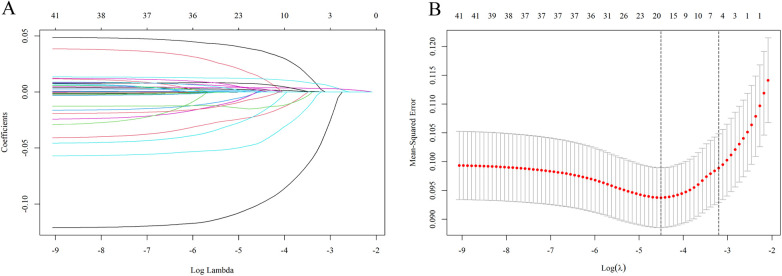
Variable screening based on lasso regression. **(A)** Variation features of variable coefficients. **(B)** The optimal value of the parameter λ in the Lasso regression model is selected through the cross—validation method.

LASSO regression was used to screen relevant features in the training set, with the coefficients of the variables displayed in Figure 2. A ten-fold cross-validation method was employed for iterative analysis. The optimal lambda value with the minimum error was selected, with log(λ) = 0.011. Ultimately, 17 variables closely associated with in-hospital mortality in septic myocardial injury were included in the model: admission age, gender, heart failure, hypertension, diabetes, CKD, liver disease, vasopressor use, SIRS score, SOFA score, APS III score, cTnt, lactate, RBC, albumin, ALT, and PTT.

### Model performance comparison

**Figure 3:**
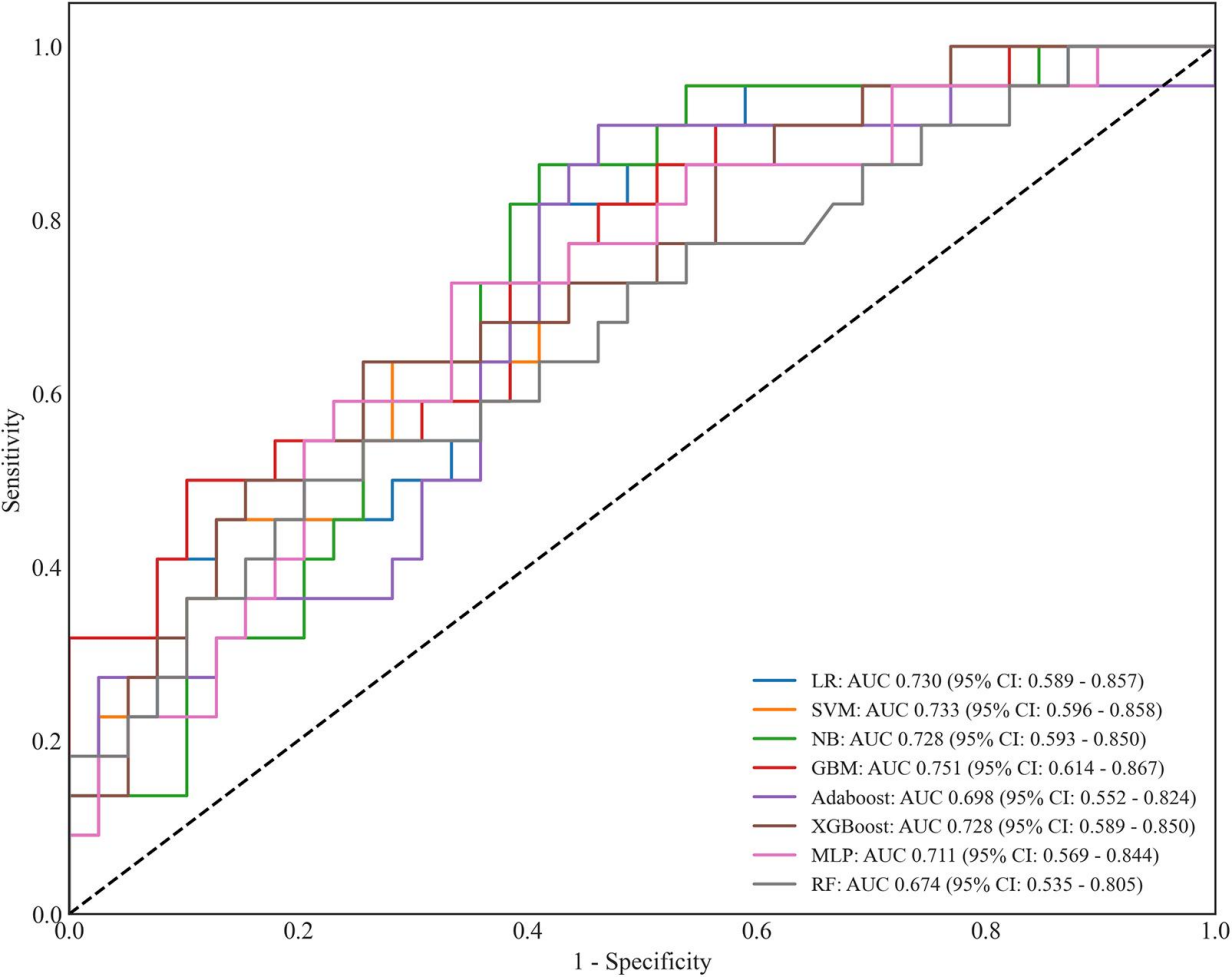
ROC curves for eight machine learning models. LR, logistic regression; SVM, support vector machine; NB, naive Bayes; XGBoost, extreme gradient boosting; GBM, gradient boosting machine; Adaboost, adaptive boosting; MLP, multilayer perceptron; RF, random forest.

We constructed eight machine learning models to identify the risk of in-hospital mortality in patients with septic myocardial injury. Figure 3 shows the discriminative performance of these models in terms of ROC. All eight models demonstrated good predictive performance for in-hospital mortality in patients with septic myocardial injury, with the GBM model performing the best. The GBM model achieved an AUC of 0.751 (95% CI: 0.614–0.867). The SVM model followed closely with an AUC of 0.733 (95% CI: 0.596–0.858), and the LR model ranked third with an AUC of 0.730 (95% CI: 0.589–0.857). The predictive performance of these models was comparable to that of the GBM model and superior to the other algorithms. Although the other models also demonstrated good predictive ability, their performance in descending order was as follows: XGBoost model (AUC = 0.728, 95% CI: 0.589–0.850), NB model (AUC = 0.728, 95% CI: 0.593–0.850), MLP model (AUC = 0.711, 95% CI: 0.569–0.844), Adaboost model (AUC = 0.698, 95% CI: 0.552–0.824), and RF model (AUC = 0.674, 95% CI: 0.535–0.805). Table 2 presents the detailed performance metrics of these eight models. The GBM model demonstrated good overall performance (sensitivity: 0.500, specificity: 0.872, F1 score: 0.579, accuracy: 0.738, recall: 0.500). Comparison with traditional mortality scoring systems was additionally performed, and the results showed that the SOFA score achieved an AUC of 0.678 (95% CI: 0.659–0.753) and the APS III score an AUC of 0.713 (95% CI: 0.678–0.835) for predicting in-hospital mortality in SIMI patients, lower than our GBM model (AUC = 0.751, 95% CI: 0.614–0.867).

**Table 2:** The performance of machine learning models for predicting in-hospital mortality in patients with septic myocardial injury.

Model	Accuracy	Specificity	Sensitivity	Precision	Recall	F1 score	AUC
LR	0.738	0.923	0.409	0.75	0.409	0.529	0.73
SVM	0.689	0.872	0.364	0.615	0.364	0.457	0.733
NB	0.639	0.821	0.318	0.500	0.318	0.389	0.728
GBM	0.738	0.872	0.500	0.688	0.500	0.579	0.751
Adaboost	0.656	0.821	0.364	0.533	0.364	0.432	0.698
XGBoost	0.721	0.846	0.500	0.647	0.500	0.564	0.728
MLP	0.672	0.821	0.409	0.562	0.409	0.474	0.711
RF	0.689	0.846	0.409	0.600	0.409	0.486	0.674

**Figure 4:**
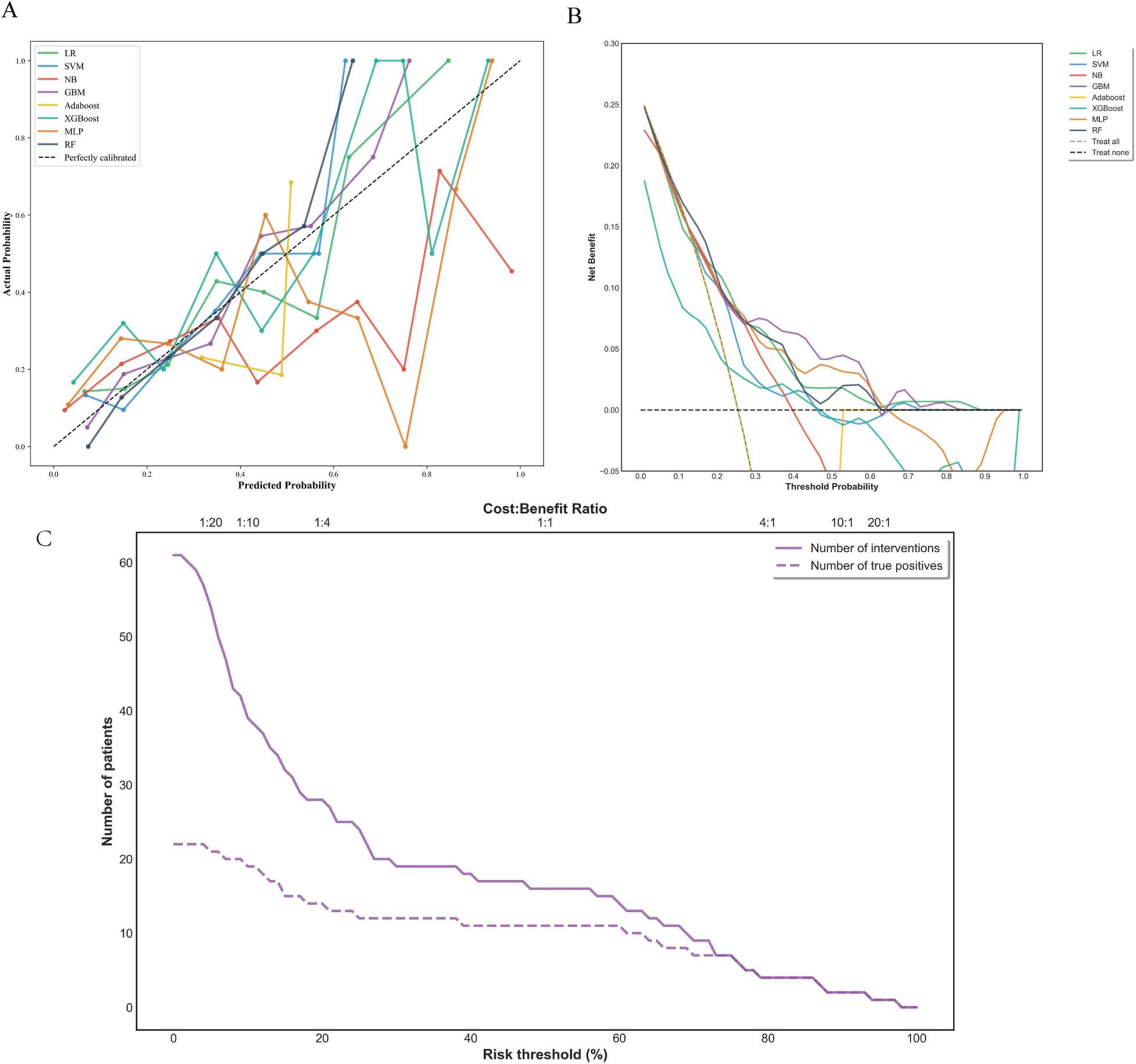
Calibration capability and clinical beneft of the model. **(A)** Calibration curve. **(B)** Decision curve analysis. **(C)** Clinical Impact Curve.

Figure 4A shows the calibration curves of the eight models, indicating good consistency between predicted probabilities and actual observations for most models. Calibration of the GBM model was formally assessed using the Hosmer-Lemeshow goodness-of-fit test and the Brier score. In the internal validation set, the Hosmer-Lemeshow test yielded a chi-square of 8.24 (*P* = 0.414), indicating no significant deviation from perfect calibration. The Brier score was 0.112, reflecting good overall accuracy of predicted probabilities. In the eICU external validation cohort, the Hosmer-Lemeshow test gave a chi-square of 10.57 (*P* = 0.233) with a Brier score of 0.094. In the FAHXJMU cohort, the Hosmer-Lemeshow test gave a chi-square of 6.89 (*P* = 0.551) with a Brier score of 0.156. These results confirm that the GBM model maintains good calibration across all three populations.

In terms of clinical applicability, Figure 4B shows that each model except XGBoost demonstrated good net benefit across a wide range of threshold probabilities, with the GBM model showing the highest net benefit. Therefore, the GBM model was selected as the optimal model for predicting in-hospital mortality in patients with septic myocardial injury. To further illustrate its clinical utility, we plotted the clinical impact curve (CIC) for the GBM model (Figure 4C). At the optimal risk threshold of 72%, the number of patients identified as high risk by the model closely matched the actual number of high-risk patients, balancing sensitivity and specificity while minimizing excessive false positives.

### External validation

Despite the significant differences in baseline characteristics between the MIMIC-IV dataset and the other two datasets, our model still demonstrated strong generalization ability. As shown in Supplementary Figure S2, the ROC curves of the GBM model in the external validation sets yielded an AUC of 0.924 [95% confidence interval (CI): 0.891, 0.952] for the eICU cohort and 0.703 (95% CI: 0.573, 0.841) for the Chinese cohort. Notably, the higher AUC in the eICU cohort (0.924) compared with the internal validation AUC in MIMIC-IV (0.751) may be explained by two well-recognized differences between the databases. First, the eICU database has been characterized as having data that are “for the most part complete and plausible” (9), reflecting its prospective design for ICU research. In contrast, MIMIC-IV is derived from routine clinical electronic health records of a single tertiary care center, where missingness in physiological and laboratory measurements is more common. Second, the eICU cohort had a substantially higher in-hospital mortality rate (25.5% vs. 13.1% in MIMIC-IV). A higher event rate generally enhances a model's discriminative ability by improving risk separation. Therefore, these factors—superior data completeness in eICU and a higher event rate—plausibly contribute to the observed performance difference, rather than data leakage or overfitting. The lower AUC in the Chinese cohort (0.703) likely reflects population heterogeneity and differences in clinical practice between Chinese and Western healthcare systems.

### Interpretability analysis

**Figure 5:**
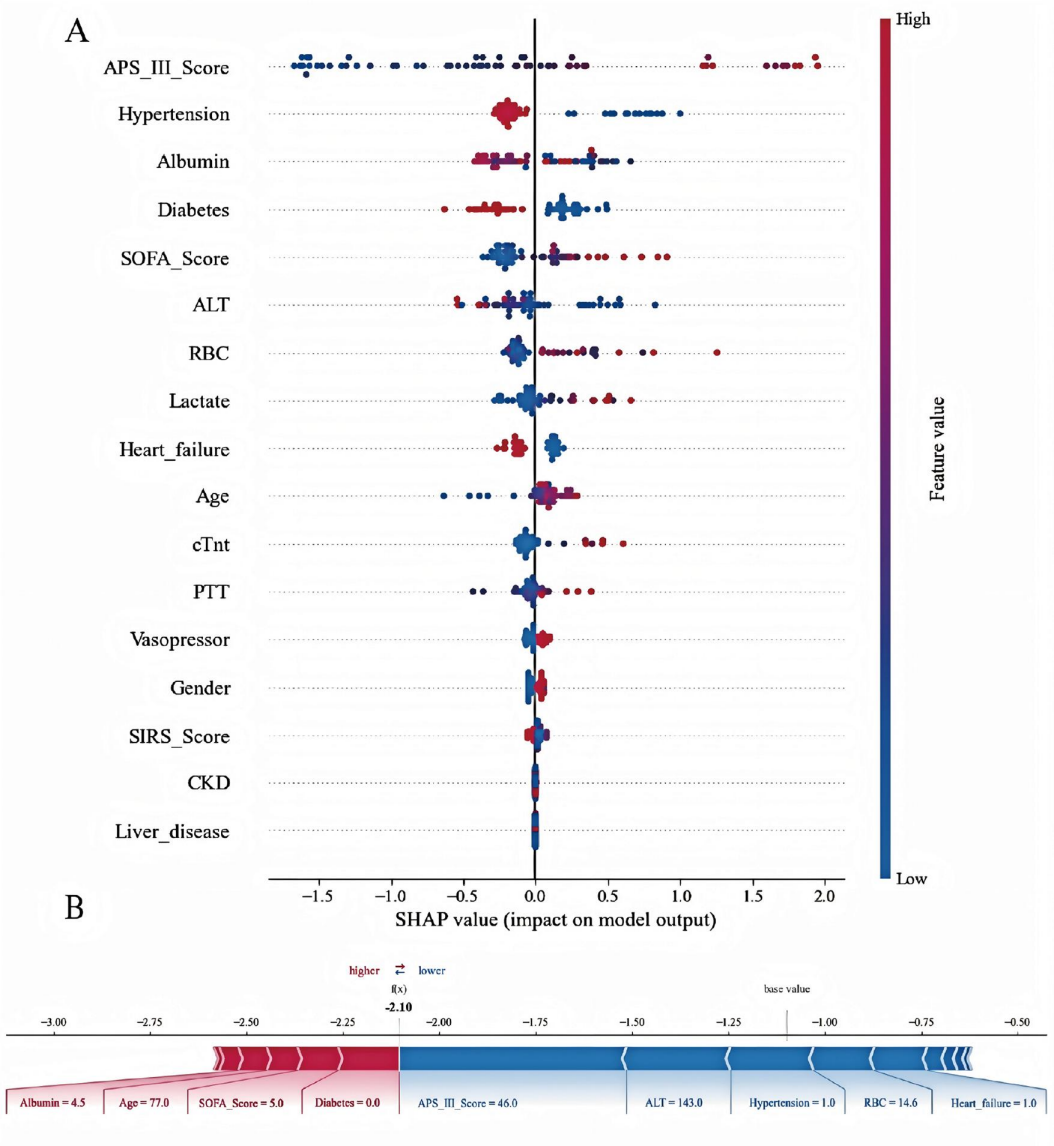
Visually interpret GBM model using SHAP. **(A)** SHAP summary point; **(B)** SHAP force plot.

Figure 5A presents a comprehensive swarm plot illustrating the variables in the GBM model. The horizontal axis represents SHAP values, while the vertical axis displays features sorted by their cumulative SHAP value impact. Each data point corresponds to a specific instance, with its position along the *x*-axis indicating the SHAP value for that particular instance and feature. APS III Score, Hypertension, Albumin, Diabetes, SOFA Score, ALT, RBC, and Lactate emerged as the eight most important factors in predicting in-hospital mortality risk in patients with SIMI. Figure 5B provides a detailed case study, demonstrating the model's prediction process for the first patient. In this visualization, red indicates higher feature values with significant impact on the prediction, while blue indicates lower feature values with significant impact. The f(x) value represents the actual SHAP value for each factor. The result indicated that, for the first patient, factors such as advanced age, a high SOFA score, and an abnormally elevated ALT contributed to an increased risk of in-hospital death. Conversely, an Albumin level of 4.5 and the absence of diabetes somewhat counterbalanced this risk.

### Application of the model

To enhance the clinical applicability of our model and facilitate rapid decision-making by clinicians, we used recursive feature elimination for refined variable selection (Supplementary Figure S3A). This optimization process allowed us to maximize model performance, with the optimal model achieving an AUC of 0.789 (95% CI: 0.669–0.893) and including five key variables: Age, SOFA Score, APS III Score, Albumin, and ALT (Supplementary Figure S3B). Furthermore, we have completed additional external validation for the simplified model in the eICU and FAHXJMU set, with AUC values of 0.922 and 0.663 respectively, (Supplementary Figure S4). To further enhance accessibility and practicality, we have deployed an optimized model on a dedicated website (https://anyuanning.shinyapps.io/SIMI/). This user-friendly platform enables clinicians to input patients' real-time indicators promptly, facilitating the assessment of in-hospital mortality risk for individuals with SIMI (Supplementary Figure S5).

## Discussion

SIMI is a common and severe complication in sepsis patients, with persistently high mortality rates. Although current guidelines emphasize the importance of early identification and intervention, existing models for predicting in-hospital mortality risk in SIMI patients still face multiple challenges. In this study, we selected 17 clinical variables through LASSO regression to construct predictive models. By comparing the predictive performance of eight machine learning algorithms, we found that the GBM model demonstrated superior performance, exhibiting not only excellent discriminative and calibration capabilities but also significant net clinical benefit. Results from external validation cohorts further confirmed the model's stability and accuracy. To deepen understanding of the model, we employed SHAP for visualization. The results revealed that eight features—APS III Score, Hypertension, Albumin, Diabetes, SOFA Score, ALT, RBC, and Lactate—contributed most significantly to the GBM model's predictions. SHAP force plots further elucidated the model's individualized prediction process for in-hospital mortality, providing comprehensive insights into its underlying mechanisms.

Current research on in-hospital mortality risk in patients with SIMI remains limited, with most studies focusing on mechanisms and risk factors for SIMI occurrence. Early identification and intervention in high-risk SIMI patients are thus critical. Patrik Johansson Blixt et al.found that mitral annular plane systolic excursion and left ventricular longitudinal wall fractional shortening are independent risk factors for septic myocardial injury (10). Serum bisphosphoglycerate mutase levels in septic patients can serve as a monitoring indicator for myocardial injury, with high levels indicating an increased risk of secondary myocardial injury events and poor outcomes (11). Ursula Müller-Werdan reported that SOFA score, left ventricular ejection fraction, ECG, and cardiac biomarkers are not helpful for grading the severity of septic cardiomyopathy (12). Meanwhile, Weiwei Qian found that procalcitonin is a sensitive biomarker for predicting myocardial injury in septic patients (13). Zheng Yang et al. observed that septic shock patients with myocardial injury exhibit exacerbated inflammatory responses during sepsis, accompanied by abnormally elevated circulating Fibroblast growth factor 23 levels (14). Additionally, aspirin has been shown to reduce mortality in SIMI patients, while dexmedetomidine improves the prognosis of septic patients with myocardial injury (15, 16).

Compared to other machine learning algorithms, the GBM model in this study demonstrated unique advantages. In complex clinical data, variable relationships often exhibit nonlinear characteristics. The GBM model effectively captures these nonlinear relationships by iteratively training multiple weak classifiers to fit intricate data patterns, thereby improving predictive accuracy. Furthermore, the GBM model exhibits greater robustness against noise and outliers. In clinical data, measurement errors and recording inaccuracies inevitably introduce noise, but the GBM model mitigates their impact on predictions, ensuring stability and reliability (17). Previous studies have shown that interpretable GBM models successfully predict persistent sepsis-associated acute kidney injury with strong performance in internal and external validation cohorts (18). This GBM model also outperformed traditional SOFA/APS III scoring systems, and its interpretability and simplified version further extended clinical utility beyond conventional risk stratification tools.

SHAP analysis in this study revealed core drivers of in-hospital mortality in SIMI patients. The SOFA score reflects the severity of multi-organ failure, while the APS III score indicates acute physiological derangements, signaling multi-organ dysfunction and critical baseline conditions. During sepsis, systemic inflammatory responses exacerbate myocardial suppression, microcirculatory dysfunction, and metabolic disturbances, accelerating organ deterioration. Lactate, a marker of tissue hypoperfusion and anaerobic metabolism, has been shown to upregulate programmed death ligand-1 in sepsis, influencing apoptosis and disease severity (19). Hypoalbuminemia not only destabilizes vascular endothelium, amplifying inflammation and fluid leakage, but also reflects nutritional depletion and hepatic dysfunction. A meta-analysis demonstrated that every 10 g/L decrease in serum albumin in critically ill patients increases in-hospital mortality by 137%, complications by 89%, and prolongs hospitalization by 72% (20). Elevated ALT indicates hepatocyte damage, impairing toxin clearance and metabolic balance (21). High RBC counts, potentially due to hemoconcentration or compensatory erythropoiesis, worsen microcirculatory dysfunction and cardiac strain. Notably, hypertension in SIMI patients may paradoxically reduce in-hospital mortality risk. This could stem from chronic hypertension-induced compensatory myocardial hypertrophy, enhancing contractility and cardiac reserve. During sepsis, this adaptive response may better sustain cardiac output and organ perfusion, mitigating circulatory failure-related mortality. Additionally, long-term antihypertensive therapies (e.g., ACE inhibitors or ARBs) may confer cardioprotective effects, attenuating SIMI (22). The association between diabetes and reduced mortality may relate to anti-inflammatory, antioxidant, and endothelial-protective effects of medications like metformin and SGLT-2 inhibitors, which alleviate myocardial injury and oxidative stress (23, 24). Chronic microvascular complications in diabetes might also trigger “ischemic preconditioning”, enhancing myocardial tolerance to hypoxia or inflammation. However, such adaptive mechanisms may be partially offset by diabetes-related comorbidities, and causal relationships require validation in large-scale studies. Notably, SOFA and APS III were included as objective indices of organ dysfunction and physiological status rather than direct mortality predictors, precluding circular reasoning; our model's integration of these scores with clinical and laboratory variables captured multidimensional prognostic information, and its predictive performance (full model AUC = 0.751, simplified model AUC = 0.789) outperformed standalone SOFA (AUC = 0.678) and APS III (AUC = 0.713), yielding incremental clinical value compared with conventional single scoring systems.

Of note, traditional myocardial injury biomarkers like cTnT showed limited contribution in our model, likely due to their ubiquitous elevation in SIMI patients, reducing discriminative power. This suggests that relying solely on cardiac biomarkers may underestimate systemic risks in SIMI patients, necessitating multi-dimensional assessment.

While this study constructed a predictive model for in-hospital mortality in septic myocardial injury patients using multi-source data, it has certain limitations. First, data from three retrospective databases (MIMIC-IV, eICU, and the First Affiliated Hospital of Xinjiang Medical University) may introduce selection bias and unmeasured confounding factors. Second, significant differences in baseline characteristics (such as comorbidities, treatment protocols, and mortality rates) between databases may lead to unstable model performance in external validation. Additionally, feature selection relying on existing variables may miss key clinical factors not included in the study, affecting prediction comprehensiveness.

## Conclusion

This study successfully developed an explainable machine learning model based on multi-center data, with the GBM algorithm demonstrating optimal performance in predicting in-hospital mortality risk in SIMI patients. The model's clinical translation through an online platform provides a tool for individualized intervention. Despite limitations such as data heterogeneity, the research findings lay an important foundation for risk stratification and mechanistic exploration of SIMI. Future studies should further optimize the predictive system through multi-center collaboration and dynamic modeling.

## Data Availability

The raw data supporting the conclusions of this article will be made available by the authors, without undue reservation.

## References

[1] Xie Y Lv H Chen D Huang P Zhou Z Wang R. A CD36-based prediction model for sepsis-induced myocardial injury. Int J Cardiol Heart Vasc. (2025) 57:101615. 10.1016/j.ijcha.2025.101615 39995812 PMC11849671

[2] Lin H Wang W Lee M Meng Q Ren H. Current status of septic cardiomyopathy: basic science and clinical progress. Front Pharmacol. (2020) 11:210. 10.3389/fphar.2020.00210 32194424 PMC7062914

[3] Salman M Cicin J Abdul Jabbar AB El-Shaer A Tauseef A Asghar N. Trends in sepsis-associated cardiovascular disease mortality in the United States, 1999 to 2022. Front Cardiovasc Med. (2024) 11:1505905. 10.3389/fcvm.2024.1505905 39717445 PMC11663846

[4] Frencken JF Donker DW Spitoni C Koster-Brouwer ME Soliman IW Ong DSY. Myocardial injury in patients with sepsis and its association with long-term outcome. Circ Cardiovasc Qual Outcomes. (2018) 11(2):e004040. 10.1161/CIRCOUTCOMES.117.004040 29378734

[5] Domenech-Briz V Gea-Caballero V Czapla M Chover-Sierra E Juárez-Vela R Santolalla Arnedo I. Importance of nutritional assessment tools in the critically ill patient: a systematic review. Front Nutr. (2022) 9:1073782. 10.3389/fnut.2022.1073782 36793999 PMC9923005

[6] Zhou PY Takeuchi A Martinez-Lopez F Ehghaghi M Wong AKC Lee EA. Benchmarking interpretability in healthcare using pattern discovery and disentanglement. Bioengineering. (2025) 12(3):308. 10.3390/bioengineering12030308 40150773 PMC11939797

[7] Johnson AEW Bulgarelli L Shen L Gayles A Shammout A Horng S. MIMIC-IV, a freely accessible electronic health record dataset. Sci Data. (2023) 10(1):1. 10.1038/s41597-022-01899-x 36596836 PMC9810617

[8] Pollard TJ Johnson AEW Raffa JD Celi LA Mark RG Badawi O. The eICU collaborative research database, a freely available multi-center database for critical care research. Sci Data. (2018) 5:180178. 10.1038/sdata.2018.178 30204154 PMC6132188

[9] O'Halloran HM Kwong K Veldhoen RA Maslove DM. Characterizing the patients, hospitals, and data quality of the eICU collaborative research database. Crit Care Med. (2020) 48(12):1737–43. 10.1097/CCM.0000000000004633 33044284

[10] Blixt PJ Nguyen M Cholley B Hammarskjöld F Toiron A Bouhemad B. Association between left ventricular systolic function parameters and myocardial injury, organ failure and mortality in patients with septic shock. Ann Intensive Care. (2024) 14(1):12. 10.1186/s13613-023-01235-5 38236316 PMC10796855

[11] Huang L Wang X Huang B Chen Y Wu X. Bisphosphoglycerate mutase predicts myocardial dysfunction and adverse outcome in sepsis: an observational cohort study. BMC Infect Dis. (2024) 24(1):173. 10.1186/s12879-024-09008-6 38326761 PMC10848385

[12] Müller-Werdan U Vogt A Werdan K. Septic cardiomyopathy-diagnosis and estimation of disease severity. Med Klin Intensivmed Notfmed. (2025) 120(3):185–91. 10.1007/s00063-024-01109-z 38345648 PMC11961453

[13] Qian W Han C Xie S Xu S. Prediction model of death risk in patients with sepsis and screening of biomarkers for prognosis of patients with myocardial injury. Heliyon. (2024) 10(5):e27209. 10.1016/j.heliyon.2024.e27209 38449610 PMC10915407

[14] Yang Z Wang J Ma J Ren D Li Z Fang K. Fibroblast growth factor 23 during septic shock and myocardial injury in ICU patients. Heliyon. (2024) 10(6):e27939. 10.1016/j.heliyon.2024.e27939 38509994 PMC10950713

[15] Dong Y Wei S Liu Y Ji X Yin X Wu Z. Aspirin is associated with improved outcomes in patients with sepsis-induced myocardial injury: an analysis of the MIMIC-IV database. J Clin Anesth. (2024) 99:111597. 10.1016/j.jclinane.2024.111597 39245010

[16] Dai X Wei H Zou D Yang Y Zhang C Chen J. Dexmedetomidine improves prognosis in septic patients with myocardial injury and lower APACHE IV scores: a retrospective cohort study. BMC Anesthesiol. (2025) 25(1):145. 10.1186/s12871-025-02906-5 40169986 PMC11959799

[17] Lee HD Nam KH Shin CM Lee HS Chang YH Yoon H. Development and validation of models to predict lymph node metastasis in early gastric cancer using logistic regression and gradient boosting machine methods. Cancer Res Treat. (2023) 55(4):1240–9. 10.4143/crt.2022.1330 36960625 PMC10582533

[18] Jiang W Zhang Y Weng J Song L Liu S Li X. An explainable machine-learning model for predicting persistent sepsis associated acute kidney injury: development, validation, and comparison with CCL14. J Med Internet Res. (2025):e62932. 10.2196/62932 40200699 PMC12070005

[19] Llibre A Kucuk S Gope A Certo M Mauro C. Lactate: a key regulator of the immune response. Immunity. (2025) 58(3):535–54. 10.1016/j.immuni.2025.02.008 40073846

[20] Yu YT Liu J Hu B Wang RL Yang XH Shang XL. Expert consensus on the use of human serum albumin in critically ill patients. Chin Med J. (2021) 134(14):1639–54. 10.1097/CM9.0000000000001661 34397592 PMC8318641

[21] Allam J Rockey DC. Aminotransferase levels in clinical practice—what is normal? Curr Opin Gastroenterol. (2025). 10.1097/MOG.0000000000001094 40227983

[22] Sobhani Shahri S Pirayesh Z Zare Noughabi A Heshmati M Khosravi Bizhaem S Jafari S. Assessing the application of American Heart Association (AHA) guidelines in the management of heart failure with reduced ejection fraction. Egypt Heart J. (2025) 77(1):28. 10.1186/s43044-025-00629-z 40063170 PMC11893950

[23] Zhu H Jia Z Li YR Danelisen I. Molecular mechanisms of action of metformin: latest advances and therapeutic implications. Clin Exp Med. (2023) 23(7):2941–51. 10.1007/s10238-023-01051-y 37016064 PMC10072049

[24] Preda A Montecucco F Carbone F Camici GG Lüscher TF Kraler S. SGLT2 Inhibitors: from glucose-lowering to cardiovascular benefits. Cardiovasc Res. (2024) 120(5):443–60. 10.1093/cvr/cvae047 38456601 PMC12001887

