# Supplementary material for: Interpretable gradient boosting machine model for predicting in-hospital mortality in sepsis-induced myocardial injury: a multicenter development, validation, and web-based clinical implementation
Source: Front Cardiovasc Med. 2026 Jul 1;13:1737106. doi: 10.3389/fcvm.2026.1737106 (PMC13369232; doi:10.3389/fcvm.2026.1737106)
Supplement: Supplementary file 1 [file Datasheet1.docx]

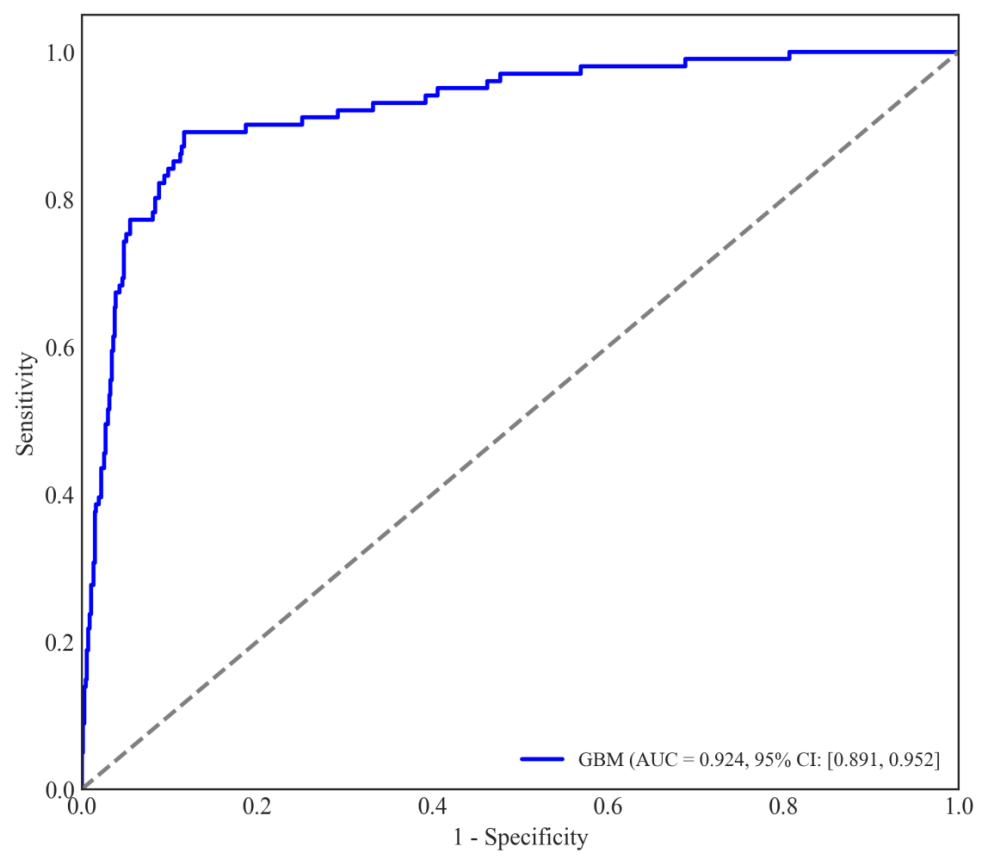


Supplementary Figure 1: The ROC curve of the GBM model on the external validation set


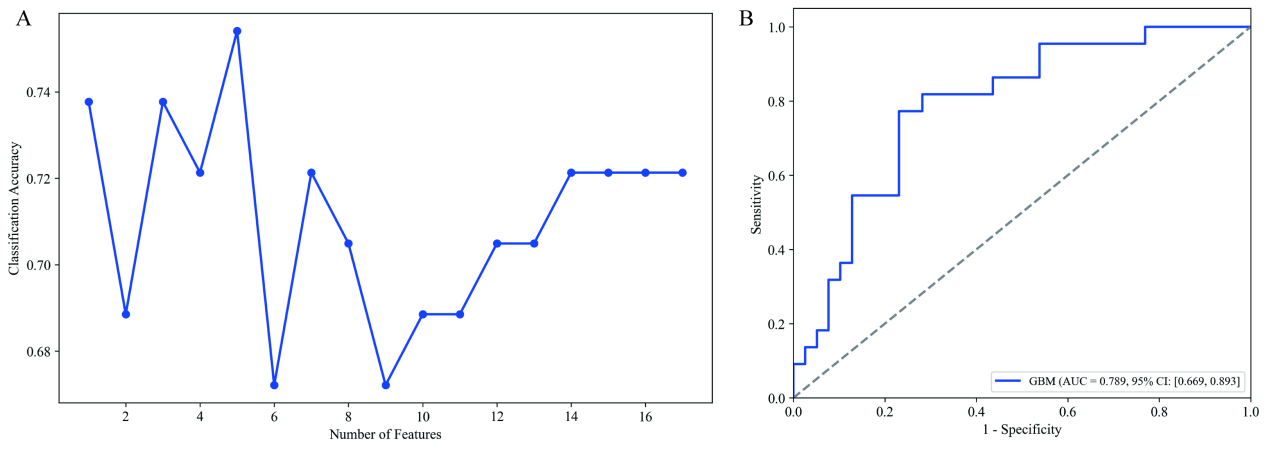


Supplementary Figure 2: Feature recursive elimination filtering variables and model performance. A. Recursive feature elimination; B.ROC curve


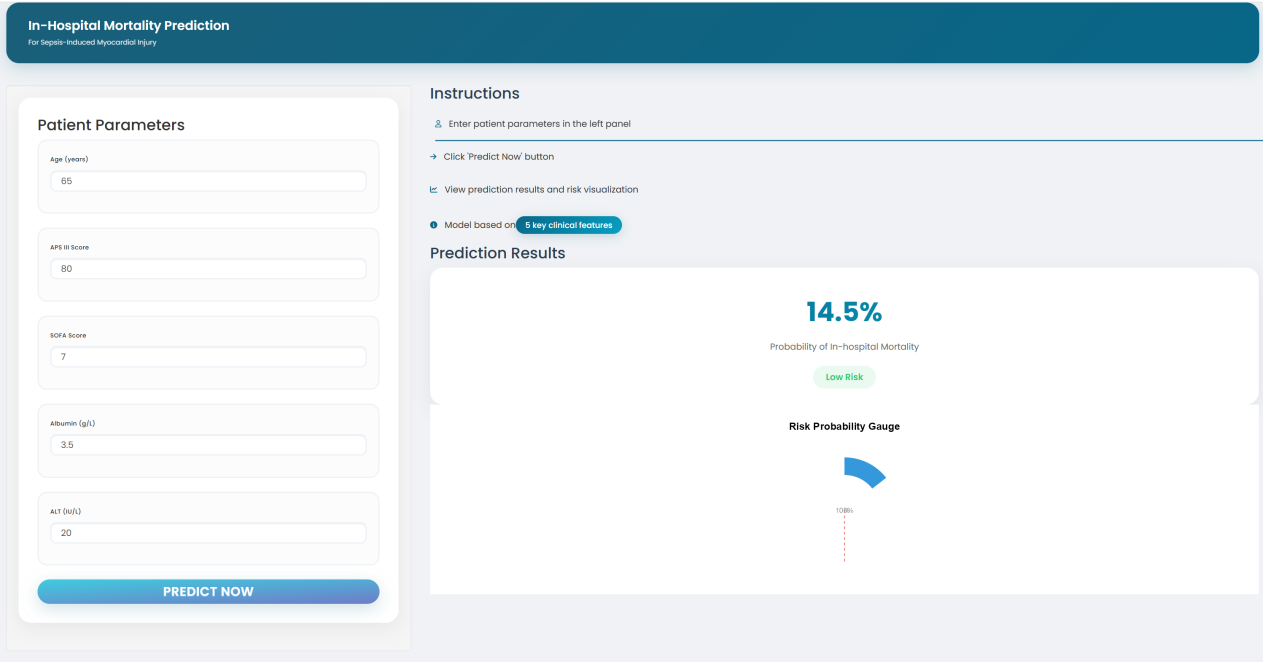


Supplementary Figure 3: User Interface of the Prediction Model
