# Supplementary material for: Interpretable gradient boosting machine model for predicting in-hospital mortality in sepsis-induced myocardial injury: a multicenter development, validation, and web-based clinical implementation
Source: Front Cardiovasc Med. 2026 Jul 1;13:1737106. doi: 10.3389/fcvm.2026.1737106 (PMC13369232; doi:10.3389/fcvm.2026.1737106)
Supplement: Supplementary file 2 [file Table1.docx]

Supplementary Table 1 Hyperparameters tuning and optimal values for the eight machine learning models

| Model | Hyperparameters Tuned | Optimal Values |
| --- | --- | --- |
| GBM | n_estimators, max_depth, learning_rate, min_samples_split, subsample | n_estimators=120, max_depth=4, learning_rate=0.08, min_samples_split=4, subsample=0.9 |
| XGBoost | n_estimators, max_depth, learning_rate, subsample, colsample_bytree | n_estimators=150, max_depth=3, learning_rate=0.09, subsample=0.7, colsample_bytree=0.9 |
| Random Forest | n_estimators, max_depth, min_samples_split, min_samples_leaf | n_estimators=200, max_depth=6, min_samples_split=3, min_samples_leaf=2 |
| AdaBoost | n_estimators, learning_rate | n_estimators=80, learning_rate=0.8 |
| SVM | kernel, C, gamma | kernel='rbf', C=0.8, gamma=0.1 |
| Logistic Regression | penalty, C, solver | penalty='l2', C=0.5, solver='saga' |
| MLP | hidden_layer_sizes, activation, learning_rate_init | hidden_layer_sizes=(64,), activation='relu', learning_rate_init=0.005 |
| Naive Bayes | - | Default parameters (GaussianNB) |

Supplementary Table 2 Baseline comparison of patients with EICU and MIMIC-IV subset

| Variables | Total (n = 1490) | EICU (n = 721) | MIMIC (n = 769) | *P* |
| --- | --- | --- | --- | --- |
|  |  |  |  |  |
| Age, M (Q₁, Q₃) | 72.00 (61.00, 81.00) | 72.00 (62.00, 81.00) | 72.00 (61.00, 81.00) | 0.615 |
| Gender, n(%) |  |  |  | 0.141 |
| Female | 659 (44.23) | 333 (46.19) | 326 (42.39) |  |
| Male | 831 (55.77) | 388 (53.81) | 443 (57.61) |  |
| Weight, M (Q₁, Q₃) | 78.45 (65.40, 94.50) | 80.00 (65.40, 99.40) | 77.00 (65.00, 90.50) | **0.002** |
| CAD, n(%) |  |  |  | **<0.001** |
| No | 1050 (70.47) | 694 (96.26) | 356 (46.29) |  |
| Yes | 440 (29.53) | 27 (3.74) | 413 (53.71) |  |
| Heart failure, n(%) |  |  |  | **<0.001** |
| No | 955 (64.09) | 647 (89.74) | 308 (40.05) |  |
| Yes | 535 (35.91) | 74 (10.26) | 461 (59.95) |  |
| Atrial fibrillation, n(%) |  |  |  | **<0.001** |
| No | 1024 (68.72) | 633 (87.79) | 391 (50.85) |  |
| Yes | 466 (31.28) | 88 (12.21) | 378 (49.15) |  |
| Hypertension, n(%) |  |  |  | **<0.001** |
| No | 821 (55.10) | 681 (94.45) | 140 (18.21) |  |
| Yes | 669 (44.90) | 40 (5.55) | 629 (81.79) |  |
| Diabetes, n(%) |  |  |  | **<0.001** |
| No | 1058 (71.01) | 632 (87.66) | 426 (55.40) |  |
| Yes | 432 (28.99) | 89 (12.34) | 343 (44.60) |  |
| CKD, n(%) |  |  |  | **<0.001** |
| No | 1041 (69.87) | 661 (91.68) | 380 (49.41) |  |
| Yes | 449 (30.13) | 60 (8.32) | 389 (50.59) |  |
| Chronic pulmonary, n(%) |  |  |  | **<0.001** |
| No | 1130 (75.84) | 660 (91.54) | 470 (61.12) |  |
| Yes | 360 (24.16) | 61 (8.46) | 299 (38.88) |  |
| Liver disease, n(%) |  |  |  | **<0.001** |
| No | 1318 (88.46) | 713 (98.89) | 605 (78.67) |  |
| Yes | 172 (11.54) | 8 (1.11) | 164 (21.33) |  |
| Steroid, n(%) |  |  |  | **<0.001** |
| No | 962 (64.56) | 579 (80.31) | 383 (49.80) |  |
| Yes | 528 (35.44) | 142 (19.69) | 386 (50.20) |  |
| Vasopressor, n(%) |  |  |  | **0.007** |
| No | 934 (62.68) | 427 (59.22) | 507 (65.93) |  |
| Yes | 556 (37.32) | 294 (40.78) | 262 (34.07) |  |
| Ventilation, n(%) |  |  |  | **<0.001** |
| No | 658 (44.16) | 275 (38.14) | 383 (49.80) |  |
| Yes | 832 (55.84) | 446 (61.86) | 386 (50.20) |  |
| RRT, n(%) |  |  |  | 0.211 |
| No | 1286 (86.31) | 614 (85.16) | 672 (87.39) |  |
| Yes | 204 (13.69) | 107 (14.84) | 97 (12.61) |  |
| SIRS Score, n(%) |  |  |  | **<0.001** |
| 0 | 174 (11.68) | 165 (22.88) | 9 (1.17) |  |
| 1 | 328 (22.01) | 258 (35.78) | 70 (9.10) |  |
| 2 | 461 (30.94) | 296 (41.05) | 165 (21.46) |  |
| 3 | 311 (20.87) | 2 (0.28) | 309 (40.18) |  |
| 4 | 216 (14.50) | 0 (0.00) | 216 (28.09) |  |
| SOFA Score, M (Q₁, Q₃) | 4.00 (3.00, 6.00) | 5.00 (4.00, 6.00) | 4.00 (2.00, 6.00) | **<0.001** |
| APS III Score, M (Q₁, Q₃) | 54.00 (42.00, 72.00) | 61.00 (46.00, 80.00) | 50.00 (40.00, 64.00) | **<0.001** |
| SAPS II Score, M (Q₁, Q₃) | 21.00 (5.00, 40.00) | 5.00 (5.00, 8.00) | 39.00 (31.00, 49.00) | **<0.001** |
| HR, M (Q₁, Q₃) | 91.00 (78.00, 107.00) | 96.00 (80.00, 112.00) | 87.00 (75.00, 100.00) | **<0.001** |
| Temperature, M (Q₁, Q₃) | 36.70 (36.20, 37.30) | 36.80 (36.40, 37.40) | 36.60 (36.00, 37.10) | **<0.001** |
| PaCO2, M (Q₁, Q₃) | 40.00 (33.00, 47.00) | 37.20 (31.00, 47.00) | 41.00 (36.00, 47.00) | **<0.001** |
| PaO2, M (Q₁, Q₃) | 103.00 (70.00, 197.75) | 95.40 (70.00, 165.00) | 111.00 (70.00, 229.00) | **<0.001** |
| Ctnt, M (Q₁, Q₃) | 0.10 (0.04, 0.29) | 0.07 (0.03, 0.19) | 0.14 (0.05, 0.49) | **<0.001** |
| Lactate, M (Q₁, Q₃) | 1.90 (1.30, 3.20) | 2.50 (1.50, 4.20) | 1.60 (1.20, 2.40) | **<0.001** |
| WBC, M (Q₁, Q₃) | 11.00 (7.20, 16.60) | 14.05 (9.60, 19.56) | 8.80 (6.50, 12.40) | **<0.001** |
| Neutrophils, M (Q₁, Q₃) | 80.05 (69.00, 87.40) | 82.60 (73.00, 88.30) | 77.20 (65.60, 85.90) | **<0.001** |
| Lymphocytes, M (Q₁, Q₃) | 10.00 (5.10, 18.70) | 7.00 (4.00, 11.50) | 14.20 (8.00, 23.90) | **<0.001** |
| Hematocrit, M (Q₁, Q₃) | 16.10 (11.90, 34.50) | 35.00 (29.80, 39.30) | 12.00 (10.60, 13.60) | **<0.001** |
| RBC, M (Q₁, Q₃) | 12.70 (3.78, 14.50) | 3.74 (3.21, 4.33) | 14.50 (13.70, 15.90) | **<0.001** |
| PLT, M (Q₁, Q₃) | 222.00 (160.00, 296.75) | 209.00 (146.00, 284.00) | 234.00 (173.00, 305.00) | **<0.001** |
| Albumin, M (Q₁, Q₃) | 3.30 (2.80, 3.80) | 3.10 (2.60, 3.50) | 3.60 (3.00, 4.00) | **<0.001** |
| Scr, M (Q₁, Q₃) | 1.50 (1.00, 2.60) | 2.04 (1.20, 3.44) | 1.20 (0.90, 1.80) | **<0.001** |
| Bilirubin, M (Q₁, Q₃) | 0.50 (0.40, 0.90) | 0.60 (0.40, 1.10) | 0.50 (0.30, 0.80) | **<0.001** |
| BUN, M (Q₁, Q₃) | 30.00 (19.00, 48.00) | 36.00 (24.00, 57.00) | 26.00 (17.00, 39.00) | **<0.001** |
| Glucose, M (Q₁, Q₃) | 20.59 (6.72, 140.00) | 142.00 (109.00, 193.00) | 6.83 (5.49, 9.60) | **<0.001** |
| ALT, M (Q₁, Q₃) | 62.00 (26.00, 97.00) | 25.00 (15.00, 47.00) | 86.00 (66.00, 120.00) | **<0.001** |
| AST, M (Q₁, Q₃) | 31.00 (21.00, 57.00) | 34.00 (22.00, 69.00) | 27.00 (20.00, 46.00) | **<0.001** |
| PT, M (Q₁, Q₃) | 15.00 (13.30, 18.20) | 16.30 (14.30, 21.00) | 13.90 (12.90, 16.00) | **<0.001** |
| PTT, M (Q₁, Q₃) | 32.00 (27.20, 38.88) | 35.00 (30.00, 42.80) | 29.40 (25.90, 34.70) | **<0.001** |
| INR, M (Q₁, Q₃) | 1.80 (1.30, 2.20) | 1.34 (1.18, 1.80) | 2.00 (1.80, 2.30) | **<0.001** |
| Hospital Death, n(%) |  |  |  | **<0.001** |
| No | 1205 (80.87) | 537 (74.48) | 668 (86.87) |  |
| Yes | 285 (19.13) | 184 (25.52) | 101 (13.13) |  |

Supplementary Table 3 Baseline comparison of patients with FAHXJMU and MIMIC-IV subset

| Variables | Total (n = 876) | FAHXJMU (n = 107) | MIMIC (n = 769) | *P* |
| --- | --- | --- | --- | --- |
|  |  |  |  |  |
| Age, M (Q₁, Q₃) | 71.00 (59.00, 81.00) | 63.00 (55.50, 74.00) | 72.00 (61.00, 81.00) | **<0.001** |
| Gender, n(%) |  |  |  | **0.001** |
| Female | 354 (40.41) | 28 (26.17) | 326 (42.39) |  |
| Male | 522 (59.59) | 79 (73.83) | 443 (57.61) |  |
| CAD, n(%) |  |  |  | **<0.001** |
| No | 446 (50.91) | 90 (84.11) | 356 (46.29) |  |
| Yes | 430 (49.09) | 17 (15.89) | 413 (53.71) |  |
| Heart failure, n(%) |  |  |  | **<0.001** |
| No | 328 (37.44) | 20 (18.69) | 308 (40.05) |  |
| Yes | 548 (62.56) | 87 (81.31) | 461 (59.95) |  |
| Atrial fibrillation, n(%) |  |  |  | **<0.001** |
| No | 487 (55.59) | 96 (89.72) | 391 (50.85) |  |
| Yes | 389 (44.41) | 11 (10.28) | 378 (49.15) |  |
| Hypertension, n(%) |  |  |  | **<0.001** |
| No | 204 (23.29) | 64 (59.81) | 140 (18.21) |  |
| Yes | 672 (76.71) | 43 (40.19) | 629 (81.79) |  |
| Diabetes, n(%) |  |  |  | **<0.001** |
| No | 506 (57.76) | 80 (74.77) | 426 (55.40) |  |
| Yes | 370 (42.24) | 27 (25.23) | 343 (44.60) |  |
| CKD, n(%) |  |  |  | **<0.001** |
| No | 452 (51.60) | 72 (67.29) | 380 (49.41) |  |
| Yes | 424 (48.40) | 35 (32.71) | 389 (50.59) |  |
| Chronic pulmonary, n(%) |  |  |  | **<0.001** |
| No | 565 (64.50) | 95 (88.79) | 470 (61.12) |  |
| Yes | 311 (35.50) | 12 (11.21) | 299 (38.88) |  |
| Liver disease, n(%) |  |  |  | **0.046** |
| No | 680 (77.63) | 75 (70.09) | 605 (78.67) |  |
| Yes | 196 (22.37) | 32 (29.91) | 164 (21.33) |  |
| Steroid, n(%) |  |  |  | **<0.001** |
| No | 463 (52.85) | 80 (74.77) | 383 (49.80) |  |
| Yes | 413 (47.15) | 27 (25.23) | 386 (50.20) |  |
| Vasopressor, n(%) |  |  |  | **<0.001** |
| No | 538 (61.42) | 31 (28.97) | 507 (65.93) |  |
| Yes | 338 (38.58) | 76 (71.03) | 262 (34.07) |  |
| Ventilation, n(%) |  |  |  | 0.338 |
| No | 431 (49.20) | 48 (44.86) | 383 (49.80) |  |
| Yes | 445 (50.80) | 59 (55.14) | 386 (50.20) |  |
| RRT, n(%) |  |  |  | 0.346 |
| No | 762 (86.99) | 90 (84.11) | 672 (87.39) |  |
| Yes | 114 (13.01) | 17 (15.89) | 97 (12.61) |  |
| SIRS Score, n(%) |  |  |  | **<0.001** |
| 0 | 17 (1.94) | 8 (7.48) | 9 (1.17) |  |
| 1 | 79 (9.02) | 9 (8.41) | 70 (9.10) |  |
| 2 | 217 (24.77) | 52 (48.60) | 165 (21.46) |  |
| 3 | 347 (39.61) | 38 (35.51) | 309 (40.18) |  |
| 4 | 216 (24.66) | 0 (0.00) | 216 (28.09) |  |
| SOFA Score, M (Q₁, Q₃) | 4.00 (3.00, 7.00) | 10.00 (8.00, 12.00) | 4.00 (2.00, 6.00) | **<0.001** |
| APS III Score, M (Q₁, Q₃) | 47.00 (34.00, 62.00) | 2.00 (2.00, 3.00) | 50.00 (40.00, 64.00) | **<0.001** |
| SAPS II Score, M (Q₁, Q₃) | 37.00 (27.00, 47.00) | 12.00 (9.00, 16.00) | 39.00 (31.00, 49.00) | **<0.001** |
| HR, M (Q₁, Q₃) | 88.00 (77.00, 103.00) | 105.00 (88.00, 122.00) | 87.00 (75.00, 100.00) | **<0.001** |
| Temperature, M (Q₁, Q₃) | 36.60 (36.10, 37.20) | 37.20 (36.70, 38.20) | 36.60 (36.00, 37.10) | **<0.001** |
| PaCO2, M (Q₁, Q₃) | 40.00 (34.00, 47.00) | 33.00 (28.00, 38.50) | 41.00 (36.00, 47.00) | **<0.001** |
| PaO2, M (Q₁, Q₃) | 108.00 (74.00, 206.00) | 98.00 (82.00, 125.50) | 111.00 (70.00, 229.00) | **0.043** |
| Ctnt, M (Q₁, Q₃) | 0.13 (0.04, 0.48) | 0.07 (0.01, 0.31) | 0.14 (0.05, 0.49) | **<0.001** |
| Lactate, M (Q₁, Q₃) | 1.60 (1.20, 2.40) | 1.60 (1.20, 2.60) | 1.60 (1.20, 2.40) | 0.511 |
| WBC, M (Q₁, Q₃) | 9.00 (6.60, 12.83) | 11.75 (7.64, 16.41) | 8.80 (6.50, 12.40) | **<0.001** |
| Neutrophils, M (Q₁, Q₃) | 73.60 (59.38, 84.73) | 9.25 (6.11, 14.12) | 77.20 (65.60, 85.90) | **<0.001** |
| Lymphocytes, M (Q₁, Q₃) | 12.40 (5.77, 22.70) | 0.72 (0.45, 1.27) | 14.20 (8.00, 23.90) | **<0.001** |
| Hematocrit, M (Q₁, Q₃) | 12.50 (10.80, 14.40) | 30.50 (24.40, 35.95) | 12.00 (10.60, 13.60) | **<0.001** |
| RBC, M (Q₁, Q₃) | 14.20 (13.30, 15.50) | 3.31 (2.67, 3.97) | 14.50 (13.70, 15.90) | **<0.001** |
| PLT, M (Q₁, Q₃) | 227.00 (163.00, 302.25) | 185.00 (87.50, 277.50) | 234.00 (173.00, 305.00) | **<0.001** |
| Albumin, M (Q₁, Q₃) | 3.70 (3.10, 4.20) | 25.73 (23.41, 28.27) | 3.60 (3.00, 4.00) | **<0.001** |
| Scr, M (Q₁, Q₃) | 1.40 (0.90, 2.60) | 81.25 (54.24, 155.51) | 1.20 (0.90, 1.80) | **<0.001** |
| Bilirubin, M (Q₁, Q₃) | 0.50 (0.40, 1.20) | 20.34 (15.39, 31.17) | 0.50 (0.30, 0.80) | **<0.001** |
| BUN, M (Q₁, Q₃) | 23.00 (15.00, 36.00) | 7.65 (4.70, 13.73) | 26.00 (17.00, 39.00) | **<0.001** |
| Glucose, M (Q₁, Q₃) | 7.13 (5.66, 9.71) | 8.32 (7.25, 11.00) | 6.83 (5.49, 9.60) | **<0.001** |
| ALT, M (Q₁, Q₃) | 85.00 (62.00, 118.00) | 39.19 (18.67, 101.03) | 86.00 (66.00, 120.00) | **<0.001** |
| AST, M (Q₁, Q₃) | 29.00 (21.00, 52.00) | 47.56 (30.73, 93.71) | 27.00 (20.00, 46.00) | **<0.001** |
| PT, M (Q₁, Q₃) | 13.85 (12.80, 16.10) | 13.80 (12.50, 16.30) | 13.90 (12.90, 16.00) | 0.394 |
| PTT, M (Q₁, Q₃) | 29.95 (26.40, 35.20) | 33.50 (30.00, 39.00) | 29.40 (25.90, 34.70) | **<0.001** |
| INR, M (Q₁, Q₃) | 2.00 (1.70, 2.30) | 1.20 (1.11, 1.42) | 2.00 (1.80, 2.30) | **<0.001** |
| Hospital Death, n(%) |  |  |  | **0.020** |
| No | 752 (85.84) | 84 (78.50) | 668 (86.87) |  |
| Yes | 124 (14.16) | 23 (21.50) | 101 (13.13) |  |

FAHXJMU : First Affiliated Hospital of Xinjiang Medical University
